# Why 11β-HSD1 inhibitors show variable efficacy in Alzheimer's therapy: an APOE4-dependent HSD11B1 mechanism

**DOI:** 10.7150/thno.126244

**Published:** 2026-01-21

**Authors:** RaiHua Lai, FengShiun Shie, RenHua Chung, Paul WeiChe Hsu, YiChung Chen, KaHei Lam, JyhLyh Juang

**Affiliations:** 1 National Center for Geriatrics and Welfare Research, National Health Research Institutes, 35, Keyan Road, Zhunan Town, Miaoli 350401, Taiwan.; 2 Institute of Molecular and Genomic Medicine, National Health Research Institutes, 35, Keyan Road, Zhunan Town, Miaoli 350401, Taiwan.; 3 Center for Neuropsychiatric Research, National Health Research Institutes, 35, Keyan Road, Zhunan Town, Miaoli 350401, Taiwan.; 4 Institute of Population Health Science, National Health Research Institutes, 35, Keyan Road, Zhunan Town, Miaoli 350401, Taiwan.; 5 Institute of Biochemistry and Molecular Biology, China Medical University, No. 100, Section 1, Jingmao Road, Taichung 406040, Taiwan.

**Keywords:** APOE4, Alzheimer's disease, cortisol metabolism, entorhinal cortex, HSD11B1

## Abstract

**Rationale:** Clinical trials for Alzheimer's disease (AD) often yield inconsistent results despite promising preclinical findings. Inhibition of 11β-hydroxysteroid dehydrogenase type 1 (HSD11B1), a cortisone reductase, has demonstrated neuroprotective effects in preclinical models. However, clinical outcomes have varied. A potential explanation is the limited representation of *apolipoprotein E ε4* (*APOE4*) carriers in preclinical studies, despite evidence that *APOE4* alters stress responses and glucocorticoid regulation. We hypothesized that *APOE4* status modulates the efficacy of HSD11B1 inhibition by influencing cortisol metabolism and AD pathology.

**Methods:** We conducted a genetic association study to test whether *HSD11B1* variants are linked to plasma cortisol levels, brain atrophy, and AD risk, stratified by *APOE4* status. Postmortem human brain tissues and wild-type mice were analyzed for *HSD11B1* expression, with emphasis on the entorhinal cortex (EC). Neuroimaging data were examined to assess correlations between cortisol levels and brain volume. In cell models, recombinant APOE4 protein was tested for regulation of *HSD11B1* expression via the transcription factor C/EBPβ and its effect on neuronal cortisol production.

**Results:** We identified a functional *HSD11B1* variant associated with elevated cortisol, increased AD risk, and accelerated EC atrophy, specifically in *APOE4* carriers. *HSD11B1* was significantly upregulated in the EC of *APOE4*-positive brains. Mechanistic studies demonstrated that APOE4, but not APOE3, upregulates *HSD11B1* via C/EBPβ, thereby increasing neuronal cortisol.

**Conclusions:** These findings explain the inconsistent efficacy of 11β-HSD1 inhibitors in AD patients by revealing an APOE4-dependent activation of HSD11B1 that promotes early EC pathology. They also support genotype-guided therapeutic strategies targeting local cortisol metabolism.

## Introduction

Genetic variation substantially influences susceptibility to and progression of AD, among which the *APOE4* allele has been consistently identified as the predominant risk factor for late-onset AD. Carriers of the *APOE4* allele exhibit an increased multifaceted propensity for amyloid-β accumulation, heightened neuroinflammation, disrupted lipid metabolism, and impaired clearance of toxic proteins in the brain 1, 2. Despite the significant impact of *APOE4* on AD pathogenesis, there remains a notable lack of understanding of APOE4-associated signaling mechanisms and therapeutic strategies specifically tailored to carriers of this genotype. Current clinical interventions largely adopt a one-size-fits-all approach, overlooking the distinct molecular and cellular pathways affected by this genotype. The absence of targeted therapies not only limits treatment efficacy in *APOE4*-positive individuals but may also contribute to the inconsistent outcomes observed in clinical trials. This highlights an urgent need to develop genotype-informed therapeutic approaches that address the unique biological mechanisms associated with *APOE4* and improve outcomes for this high-risk population.

Accumulating evidence suggests that APOE4 modulates HPA-axis function, perturbs glucocorticoid receptor (GR) signaling, and confers greater stress sensitivity 3. Actually, cortisol levels in the body can be elevated through two primary mechanisms. The first mechanism entails activation of the HPA axis, leading to stress-induced cortisol secretion from the adrenal glands into the circulation. The second, more local pathway operates at the cellular level, where 11β-hydroxysteroid dehydrogenase type 1 (HSD11B1) regenerates active cortisol by reducing cortisone 4. HSD11B1 functions as a NADPH-dependent reductase primarily in metabolic and neural tissues, including the brain. In this context, HSD11B1 enables tissue-specific amplification of glucocorticoid signaling independent of systemic HPA activation, thereby playing a critical role in modulating local stress responses and potentially contributing to region-specific neuronal vulnerability. In addition to the role in glucocorticoid metabolism, emerging evidence reveal that HSD11B1 also modulates neuroinflammation and cognitive decline, making it an attractive target for AD therapy 5. However, current understanding of HSD11B1's role in local cortisol signaling in AD brain remains limited.

Emerging evidence suggests that the long-term elevation of glucocorticoids increases the risk and accelerate the progression of AD 6, 7. *APOE4* carriers exhibit early pathological stress and dysregulated cellular events during MCI, which may be more amenable to therapeutic intervention than the widespread neurodegeneration seen in advanced AD. Although cortisol functions as a stress hormone in the context of AD, the mechanistic link between *APOE4*, cortisol activation, and the brain subregions affected in early AD remains incompletely understood.

In this study, we focus on the response of EC to dysregulated cortisol signaling in AD. The EC lies in the medial temporal lobe, immediately rostral to the hippocampus, acts as a key interface between the neocortex and hippocampus 8. *APOE4*-related changes in the EC are detectable even in youth, and EC atrophy is one of the earliest structural hallmarks of AD 9, 10, raising the question of whether APOE4 engages EC-specific pathways. Supporting a direct role for stress hormones, glucocorticoids rapidly alter microcircuit activity in layer II of the medial EC, reducing inhibitory synaptic events and enhancing α1-adrenergic responses in principal cells 11. Anatomical studies show high levels of GR mRNA and protein in the adult EC 12, and EC manipulation can influence hippocampal GR expression 13, highlighting tight functional coupling within this network. Together, these findings suggest that cortisol may contribute to AD progression via its effects on EC circuitry.

Notably, convergent preclinical work shows that lowering brain 11β-HSD1 activity reduces local glucocorticoid amplification and improves memory and neuropathology 14, 15. In contrast, randomized clinical trials in AD have been largely negative at primary endpoints; for example, ABT-384 did not improve cognition versus placebo 16, although biomarker enriched analyses suggest possible signals, such as with Xanamem in participants with elevated plasma pTau181 17. This gap between preclinical and clinical findings suggests that host modifiers of glucocorticoid tone may shape treatment response. Given that *APOE4* carriers display altered stress and glucocorticoid regulation and greater stress related cognitive vulnerability 3, 18, 19, an approach that evaluates HSD11B1 inhibition with stratification by *APOE4* status and guidance by biomarkers is biologically plausible and may improve therapeutic yield.

In this study, we aim to address the translational gap between the promising effects of 11β-HSD1 inhibition observed in preclinical models and the inconsistent outcomes seen in AD clinical trials. We hypothesize that *APOE4* status modulates the therapeutic efficacy of HSD11B1 inhibition by altering cortisol metabolism and contributing to AD pathology. To test this hypothesis, we performed a multimodal integrative analysis stratified by *APOE4* genotype, incorporating genetic association studies, neuroimaging data analysis, plasma cortisol measurements, EC atrophy assessment, and evaluation of MCI-to-AD progression. Our findings suggest that *APOE4* genotype may influence individual responses to HSD11B1 inhibitor therapy and provide mechanistic insight into an APOE4-dependent pathway involving C/EBPβ-mediated HSD11B1 upregulation and local cortisol dysregulation during early AD progression.

## Materials and Methods

### Cell lines and transfection

Mouse hippocampal neuronal cell line HT-22 (Sigma-Aldrich, SCC129) and the *APOE3* (E3/E3) genotype human neuroblastoma cell line SH-SY5Y (ATCC, CRL-2266) were cultured in Dulbecco's Modified Eagle Medium (Gibco) and Minimum Essential Medium (Gibc), respectively, at 37°C in a 5% CO₂ humidified atmosphere. APOE genotyping was performed using DNA sequencing. The primers employed for amplification and sequencing were as follows: APOE_F: 5'-GAC CAT GAA GGA GTT GAA GGC CTA C-3' and APOE_R: 5'-CTC GCG GGC CCC GGC CTG GTA-3' 20. RNA interference-mediated knockdown of C/EBPB or HSD11B1 was achieved by transiently transfecting SH-SY5Y cells with either *siC/EBPB*, *siHSD11B1* or a control siRNA (Stealth siRNA; Invitrogen) was transfected using DharmaFECT (Dharmacon).

### Primary entorhinal cortical neuron cultures

Primary entorhinal cortical neuron cultures were prepared from postnatal day 1 C57BL/6J mouse pups of either sex, as previously described 21, 22. Brains were removed and placed in Hank's balanced salt solution. The entorhinal cortex was carefully microdissected under a stereomicroscope. Dissected tissues were pooled and enzymatically dissociated using a papain-based enzyme solution, followed by incubation with a trypsin inhibitor solution. After gentle trituration, a single-cell suspension was obtained and cells were seeded at a density of 5 × 10⁴ cells per well onto 48-well plates with pre-coated poly-D-lysine and laminin. The cells were maintained at 37 °C in a humidified incubator with 5% CO₂ in serum-free Neurobasal medium supplemented with 2% B27, 1% L-glutamine (2 mM), and 1% penicillin/streptomycin. Twenty-four hours after seeding, one-third of the medium was replaced with fresh medium, followed by medium changes every 3-4 days thereafter. Neuronal enrichment of the cultures was confirmed by immunolabeling with the neuronal marker MAP-2. Experiments were conducted at 6-10 days in vitro (DIV). The animal procedures were approved by the Institutional Animal Care and Use Committee (IACUC) of the National Health Research Institutes (NHRI) under protocol number NHRI-IACUC-103136-A and NHRI-IACUC-114027.

### Cortisol ELISA assay

Culture cells were treated with VLDL (25 μg/mL; Sigma-Aldrich, LP1) and HDL (25 μg/mL; Sigma-Aldrich, LP3) in the presence of human APOE3 (10 μg/mL, Sigma-Aldrich, SRP4696) or APOE4 (10 μg/mL, Sigma-Aldrich, A3234) for 3 days, following modifications from previous literature 23. Following this treatment, cortisone (0.4 μg/mL; Sigma-Aldrich, C2755) was added to the culture medium, either alone or in combination with carbenoxolone disodium (5-15 μM; MedChemExpress, HY-B1367), for an additional 24 hours. The culture medium was then collected, and cortisol levels were measured using the Cortisol ELISA Kit (Elabscience, Cat# E-EL-0157) according to the manufacturer's instructions.

### Quantitative real-time reverse transcription-polymerase chain reaction (qRT-PCR)

qRT-PCR was performed with modifications based on previously published methods 22. The primers used were as follows: Human GAPDH-F: 5'-CCT GCC AAA TAT GAT GAC ATC AAG-3; Human GAPDH-R: 5'-ACC CTG TTG CTG TAG CCA AA-3'; Human HSD11B1-F: 5'-GTT ACG TGG TCC TGA CTG TAG C-3'; Human HSD11B1-R: 5'- GCA GCA ACC ATT GGA TAA GCC AC-3'; Human HSD11B2-F: 5'- CAA ACC AGG AGA CAT TAG CCG C-3'; Human HSD11B2-R: 5'- TCA CCT CCA TGC AGC TAC GGA A-3'; Mouse HSD11B1-F: 5'- CTC TGC TCA CTA CAT TGC TGG C-3'; Mouse HSD11B1-R: 5'- GAG ACA GCG AGG TCT GAG TGA T-3'; Mouse HSD11B2-F: 5'- CAG AGG ACA TCA GCC GTG TTC T-3'; Mouse HSD11B2-R: 5'- GAA AGT CGC CAC TGG AGA CAG T-3'; Mouse GAPDH-F: 5ʹ-CAT CAC TGC CAC CCA GAA GAC TG-3ʹ; Mouse GAPDH-R 5ʹ -ATG CCA GTG AGC TTC CCG TTC AG-3ʹ.

### Identification of potential transcriptional regulators of HSD11B1

To identify potential transcriptional regulators of *HSD11B1*, we first identified genes co-expressed with HSD11B1, we analyzed ARCHS4 RNA-seq data from the Enrichr database (https://maayanlab.cloud/Enrichr/), which integrates publicly available transcriptomic datasets. The top 50 co-expressed genes were selected for further analysis. Next, we performed enrichment analysis using the Enrichr transcription factor (TF) database and UCSC Genome Browser Position Weight Matrices (PWMs) to identify enriched TF binding motifs in its regulatory regions.

To further validate potential TF regulation, chromatin immunoprecipitation sequencing (ChIP-seq) data from the ReMap database within the UCSC Genome Browser (https://genome.ucsc.edu/) was analyzed. This analysis focused on the presence of CCAAT/enhancer-binding protein beta (C/EBPβ) motifs in the HSD11B1 promoter region. The identified binding sites were visualized to confirm the potential regulatory role of C/EBPβ in HSD11B1 transcription.

### Chromatin immunoprecipitation (ChIP) assay

Culture cells were treated with HDL (25 μg/mL) or recombinant human ApoE3 or APOE4 proteins (10 μg/mL) for 3 days and then ChIP assays were performed as described 24. Briefly, 2 × 10⁷ cells were cross-linked with 1% formaldehyde at 37°C, followed by quenching and washing with cold phosphate-buffered saline (PBS). A portion of the cells was reserved for input chromatin DNA analysis. Cells were lysed in 200 μL ChIP lysis buffer and incubated on ice. Chromatin was sonicated using a Bioruptor plus at low intensity for 10 pulses of 30 seconds each. The sonicated lysate was diluted to 2 mL with ChIP dilution buffer and precleared with 80 μL of protein A Plus agarose beads at 4°C for 1 hour. The precleared lysate was incubated overnight at 4°C with an anti-C/EBPβ antibody (4 μg), with an IgG-antibody control as a negative control. Immunocomplexes were collected using 60 μL of protein A Plus agarose beads and sequentially washed with low-salt buffer, and TE buffer. Complexes were eluted with 1% SDS and 0.1 M NaHCO₃, followed by cross-link reversal at 65°C and proteinase K digestion. DNA was extracted using phenol/chloroform, ethanol-precipitated, and resuspended in 40 μL distilled water. Finally, 1 μL of ChIP DNA was used for qPCR amplification on a QuantStudio 1 Real-Time PCR System with specific primers for HSD11B1: 5'- GAC TCG GGT AGG GAT GCT CA-3' and 5'- GCA GCC TGT GTG GTG GAT TT-3'.

### Antibody

The antibodies used for Western blotting were as follows: anti-GAPDH, GeneTex, Cat# GTX100118; anti-HSD11B1, ABClonal, Cat# A1619; anti-APOE4, Cell Signaling Technology, Cat# 8941; anti-C/EBPβ, ABclonal, A0711; anti-pC/EBPβ (T235), Cell Signaling Technology, #3084.

### Brain *HSD11B1* and *HSD11B2* expression data analysis

Brain expression data for HSD11B1 and HSD11B2 were obtained from the Allen Human Brain Atlas microarray database (https://human.brain-map.org/microarray/), using probes A_23_P63209 and A_23_P14986, respectively. The dataset comprises genome-wide microarray expression profiles from six neurologically normal adult donors (aged 24-57 years; five males and one female), covering multiple anatomically defined brain regions. To represent the entorhinal cortex (EC), samples from the lateral bank of the parahippocampal gyrus and the bank of the collateral sulcus were selected, as these regions are largely occupied by the entorhinal area 25. The hippocampus was defined to include the dentate gyrus and the CA1-CA4 subfields. Based on these anatomical definitions, HSD11B1 expression levels in the EC and hippocampus were calculated separately for each individual donor. In addition, in situ hybridization (ISH) data for HSD11B1 were retrieved from the Allen Mouse Brain Atlas (https://portal.brain-map.org/) and analyzed using experimental datasets 79762381 and 73520987.

Gene expression data for *HSD11B1* in the EC were obtained from the GSE48350 dataset on NCBI, which includes microarray expression data from human AD brains. To ensure age-matching, a total of 13 AD subjects aged 85-94 years were selected for analysis, including 9 APOE4 carriers and 4 non-carriers. For comparison, the expression levels of *HSD11B1* and *CEBPB* were analyzed between the two groups to assess the differences in expression in the EC tissues, with *GAPDH* used as an internal control.

### *HSD11B1* SNP analysis using ADNI and ROSMAP datasets

SNP data for *HSD11B1* were obtained from the ADNI_1_GWAS_Plink file within the ADNI database and from the Religious Orders Study and Memory and Aging Project (ROSMAP) cohort. Baseline demographic characteristics of the study participants are summarized in Table S1. A total of 556 participants from the ADNI cohort and 1272 participants from the ROSMAP cohort were included in the analysis. Participants were first stratified by *APOE4* status, and subsequently categorized into cognitively normal (CN) and late MCI/AD groups. Genotypic and statistical analyses were conducted using PLINK software, employing logistic regression models with adjustments for age and sex.

The association between the *HSD11B1* SNP rs2282738 and cortisol levels, EC, and hippocampal (hippo) volumes in MCI patients was evaluated. A range of variables evaluating disease progression and cognitive function were obtained from the “ADNIMERGE.csv” file provided by ADNI. These included the time from MCI to AD diagnosis, *APOE4* genotype information, and MRI brain volume data for the EC, hippocampus (Hippo), fusiform gyrus, whole brain (W) and blood cortisol level. Statistical significance was assessed using the Brown-Forsythe ANOVA test, with a threshold of p < 0.05. Ethical approval for all study protocols was granted by the Research Ethics Committee at the National Health Research Institutes (protocol no. EC1001103).

### Cortisol, APOE4, and brain changes in MCI progression

We analyzed data from the ADNI cohort to investigate how plasma cortisol levels and *APOE4* status affect brain volume changes and progression from MCI to AD. MCI participants were stratified by *APOE4* genotype and baseline plasma cortisol levels (high vs. low). EC and hippocampal volumes were assessed at baseline and follow-up (6-36 months) using MRI, normalized to total brain volume. Differences in EC volume over time were compared using ANOVA. Separately, we examined how baseline cortisol levels relate to the time to AD conversion using Kaplan-Meier survival analysis, with log-rank tests assessing group differences. MCI subjects who progressed to AD within 3 months were excluded. Analyses were performed to evaluate whether cortisol-related effects differ by *APOE4* status.

### Statistical analysis

Details on sample size, central tendency, and significance are provided in figure legends or tables. Group comparisons were conducted according to data type, using two- or one-tailed t tests for continuous variables and *χ*^2^ tests for categorical variables. One-way ANOVA was used for comparisons among multiple groups, with Brown-Forsythe ANOVA applied when variance was unequal or outliers were present. MCI-to-AD progression was analyzed using Kaplan-Meier curves and log-rank tests. A p-value < 0.05 was considered significant.

## Results

### Association of HSD11B1 polymorphisms with AD risk is *APOE4*-dependent

Although *HSD11B1* SNPs have been proposed to affect AD risk, these associations have not been consistently replicated 5. To determine whether *APOE4* modifies the genetic contribution of *HSD11B1* to AD pathogenesis, we examined the association between *HSD11B1* genetic variants and AD susceptibility, stratified by *APOE4* carrier status in two large independent clinical cohorts. Analysis of SNP array data from the ADNI cohort revealed two variants, rs2282738 and rs2282739, that were significantly associated with elevated AD risk among *APOE4* carriers, but not in non-carriers. Similarly, two different SNPs (rs7539852 and rs6672256) in the ROSMAP cohort were identified to be also significantly associated with increased AD risk in *APOE4* carriers (Table 1). We conducted linkage disequilibrium analysis and found all four SNPs were actually located within a single LD block in the EUR population. Among them, rs2282738 was selected as the tag SNP, while rs2282739, rs7539852, and rs6672256 showed strong linkage disequilibrium with rs2282738 (R² = 0.977, 0.886, and 0.886; GRCh38). This suggests that rs2282738 can be used as the representative variant for this locus in subsequent functional analyses. In contrast, no significant associations were observed among *APOE4* non-carriers in either cohort. These results support our hypothesis that the genetic influence of *HSD11B1* on AD risk may be specific to individuals carrying the *APOE4* allele.

### Predominant *HSD11B1* expression in the EC of *APOE4*-positive brains

**Figure 1:**
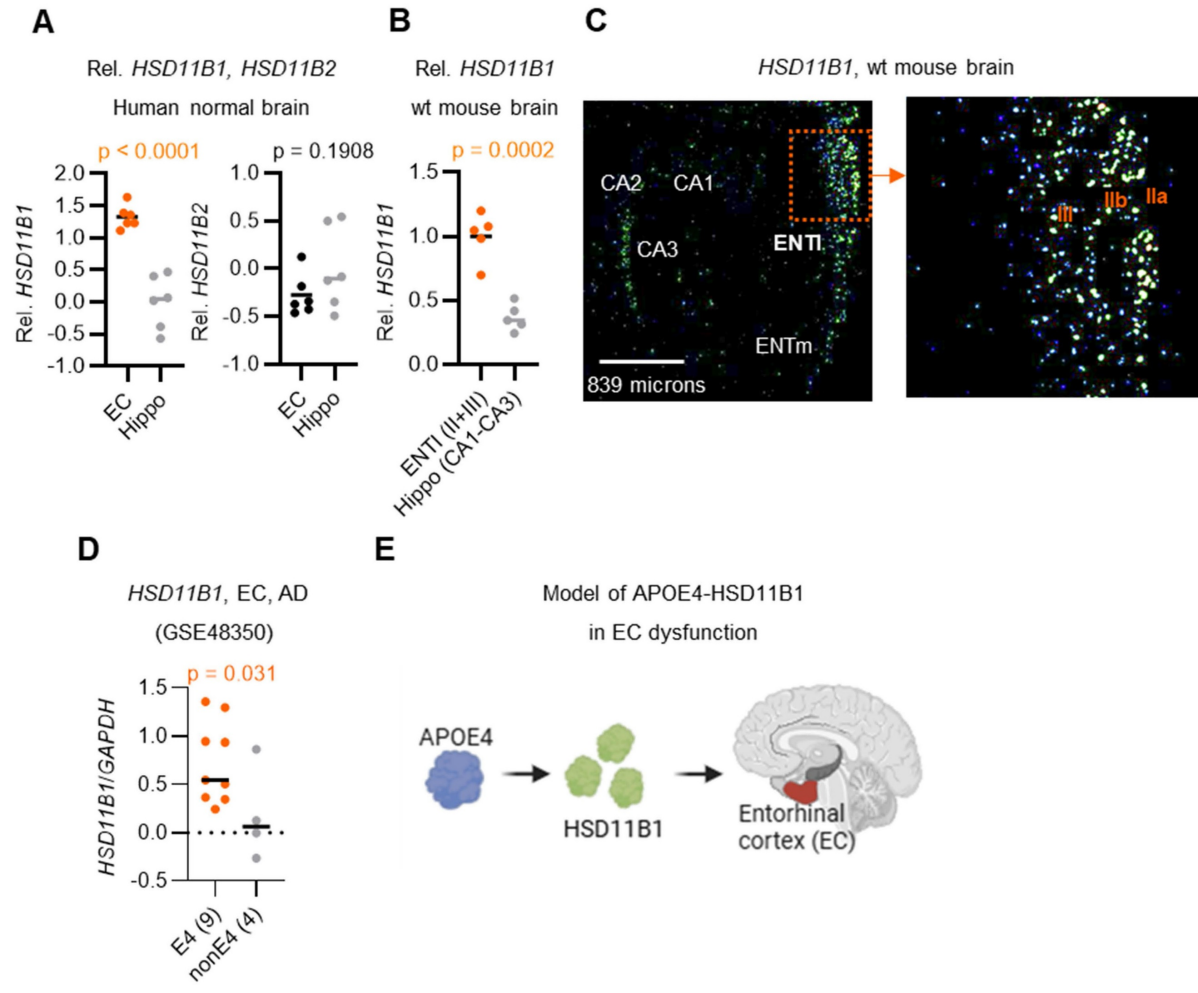
***HSD11B1* expression patterns in the EC and hippocampus in human and mouse brains. A.** Relative quantification of *HSD11B1* expression in the human hippocampus versus the entorhinal cortex (EC). Expression data were obtained from the Allen Human Brain Atlas using probe A_23_P63209 for *HSD11B1* and A_23_P14986 for HSD11B2.** B.** Relative quantification of *HSD11B1* expression from in situ hybridization (ISH). Expression levels were calculated from images captured at five distinct locations. Data were obtained from the Allen Mouse Brain Atlas (Experiment Nos. 79762381 and 73520987).** C.** Representative ISH images depicting HSD11B1 expression in mouse brain regions, including the lateral entorhinal cortex (ENTl), medial entorhinal cortex (ENTm), and the Cornu Ammonis (CA1-CA3) regions of the hippocampus. IIa, IIb, and III denote specific layers of the EC. **D.** Comparison of *HSD11B1* expression levels in the EC of human AD brains between *APOE4* carriers and non-carriers. Microarray data from EC tissues were obtained from the GSE48350 dataset, with *HSD11B1* expression normalized to *GAPDH*. Statistical significance was determined using a one-tailed t-test, based on the hypothesis that *APOE4* carriers exhibit higher *HSD11B1* and C/EBPβ expression.** E.** Schematic model of the proposed APOE4-HSD11B1 signaling pathway in promoting EC dysfunction.

Because HSD11B1 mediates local cortisol regeneration, and that brain cortisol levels are known to increase with age 26, it is important to investigate whether local cortisol is elevated in AD, particularly in the EC and hippocampus, regions among the earliest and most severely affected by AD pathology 9. To explore this, we analyzed *HSD11B1* expression patterns using publicly available data from the Allen Brain Atlas (Figure 1A, 1B). Unexpectedly, our analysis revealed that *HSD11B1* expression was significantly higher in the EC compared to the hippocampus in both normal humans (p < 0.0001) and mouse brains (p = 0.0002). In the EC, *HSD11B1* expression was highest in the lateral subdivision and was mainly detected in layer II/III cells of the mouse brain that morphologically resembled pyramidal neurons (Figure 1C, right). In contrast, *HSD11B1* expression in the hippocampus was substantially lower and was primarily confined to the CA3 subfield (Figure 1C, left; Figure S1A). This finding is consistent with prior immunohistochemical evidence indicating that AD-related pathology initially arises in layer II neurons of the EC 27-29. These findings suggest that the selective enrichment of *HSD11B1* in the EC may drive localized cortisol activation under normal conditions. Furthermore, its preferential localization to layers II and III highlights a potential mechanism by which the EC becomes particularly vulnerable to atrophy during AD progression.

Building on these findings, we next examined *HSD11B1* expression in AD patients to assess whether genetic risk factors might further influence local cortisol activation. Importantly, the individuals carrying the *APOE4* allele exhibited significantly higher levels of *HSD11B1* in the EC compared to non-*APOE4* carriers (Figure 1D). Notably, this *APOE4*-associated increase was not observed in the hippocampus (Figure S1B). These results further support our hypothesis that APOE4 may enhance local cortisol activation selectively within the EC, thereby exacerbating regional vulnerability in AD. Based on these observations, we propose a model in which APOE4 promotes *HSD11B1* expression in the EC, leading to excessive cortisol regeneration and contributing to EC dysfunction and neurodegeneration in AD brains (Figure 1E).

### Association of *HSD11B1* polymorphisms with local cortisol activation and early EC atrophy

**Figure 2:**
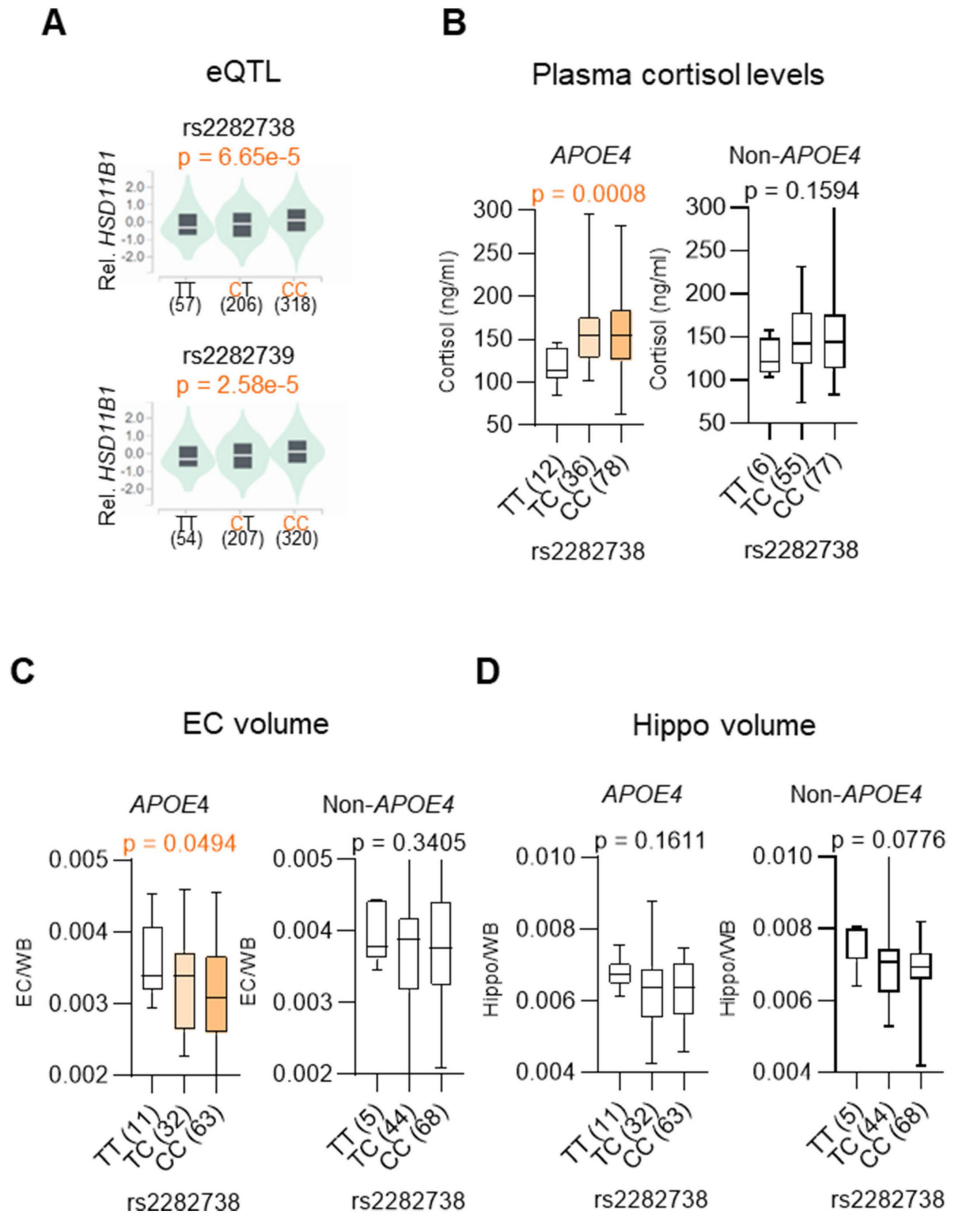
** SNPs in *HSD11B* are correlated with plasma cortisol levels and selective EC atrophy in *APOE4* MCI individuals**. **A.** eQTL violin plots displaying *HSD11B1* expression stratified by SNPs rs2282738 and rs2282739. Data were adapted from the GTEx portal, illustrating the relationship between these SNPs and *HSD11B1* expression. **B.** Association between the *HSD11B1* SNP rs2282738 and plasma cortisol levels in *APOE4* and non-*APOE4* MCI subjects, using data from the ADNI database. Medians are represented by horizontal bars and interquartile ranges by vertical bars. Statistical analysis was performed using the Brown-Forsythe ANOVA test (p < 0.05). **C, D.** Associations between the HSD11B1 SNP and EC or hippocampal (Hippo) volumes, normalized by total brain volume (WB), in MCI subjects.

Given our earlier finding that the association between *HSD11B1* and AD risk is specific to individuals carrying the *APOE4* allele, we then examined whether AD-associated *HSD11B1* SNPs represent functional variants that influence local gene expression 30. To address this, eQTL analysis was performed using data from the GTEx Portal (https://gtexportal.org/). We assessed whether AD-associated SNPs (rs2282738 and rs2282739) were associated with HSD11B1 expression. The analysis revealed significant eQTL associations for two SNPs, indicating that these variants are functionally linked to *HSD11B1* gene expression (rs2282738: p = 6.65 × 10⁻⁵; rs2282739: p = 2.58 × 10⁻⁵) (Figure 2A).

Building on these findings, we next examined whether HSD11B1 SNPs influence downstream cellular effectors in an *APOE4*-dependent manner. Notably, rs2282738 was significantly associated with plasma cortisol levels among *APOE4* carriers (p = 0.0008), but not among non-carriers (p = 0.1594) (Figure 2B). Specifically, *APOE4* carriers with the rs2282738 CC or CT genotype showed a 1.34-fold increase in plasma cortisol, compared to a modest 1.18-fold increase in individuals lacking the *APOE4* allele. These results suggest that the AD risk-associated rs2282738 variant may modulate systemic cortisol levels in a genotype-specific manner, with APOE4 potentially acting as an enhancer of this effect.

Given that elevated cortisol is linked to neurodegeneration, we further examined whether rs2282738 is associated with AD-related early brain atrophy, focusing on the EC and hippocampus. Using structural MRI data, we analyzed the correlation between rs2282738 genotypes and regional brain volumes. We observed a significant association between rs2282738 and EC volume (p = 0.0494), with a trend across TT, TC, and CC genotypes (Figure 2C), whereas no significant association was detected with hippocampal volume (Figure 2D). Although a similar trend was observed in *APOE4* hippocampal, the effect was more pronounced in EC: those with the rs2282738 CC genotype exhibited a 9% reduction in median EC volume compared with TT carriers, whereas a 5% decrease in hippocampal volume relative to TT carriers. These findings suggest that rs2282738 interacts with *APOE4* to exacerbate EC atrophy, with a comparatively stronger effect in this region than in the hippocampus.

These findings support a model in which *HSD11B1* polymorphisms contribute to AD risk by promoting cortisol dysregulation and selective vulnerability of the EC. Collectively, our genetic and imaging analyses reinforce the hypothesis that HSD11B-driven cortisol activation may underlie *APOE4*-specific susceptibility to neurodegeneration in AD.

### Elevated plasma cortisol levels predict accelerated EC atrophy and MCI-to-AD progression

**Figure 3:**
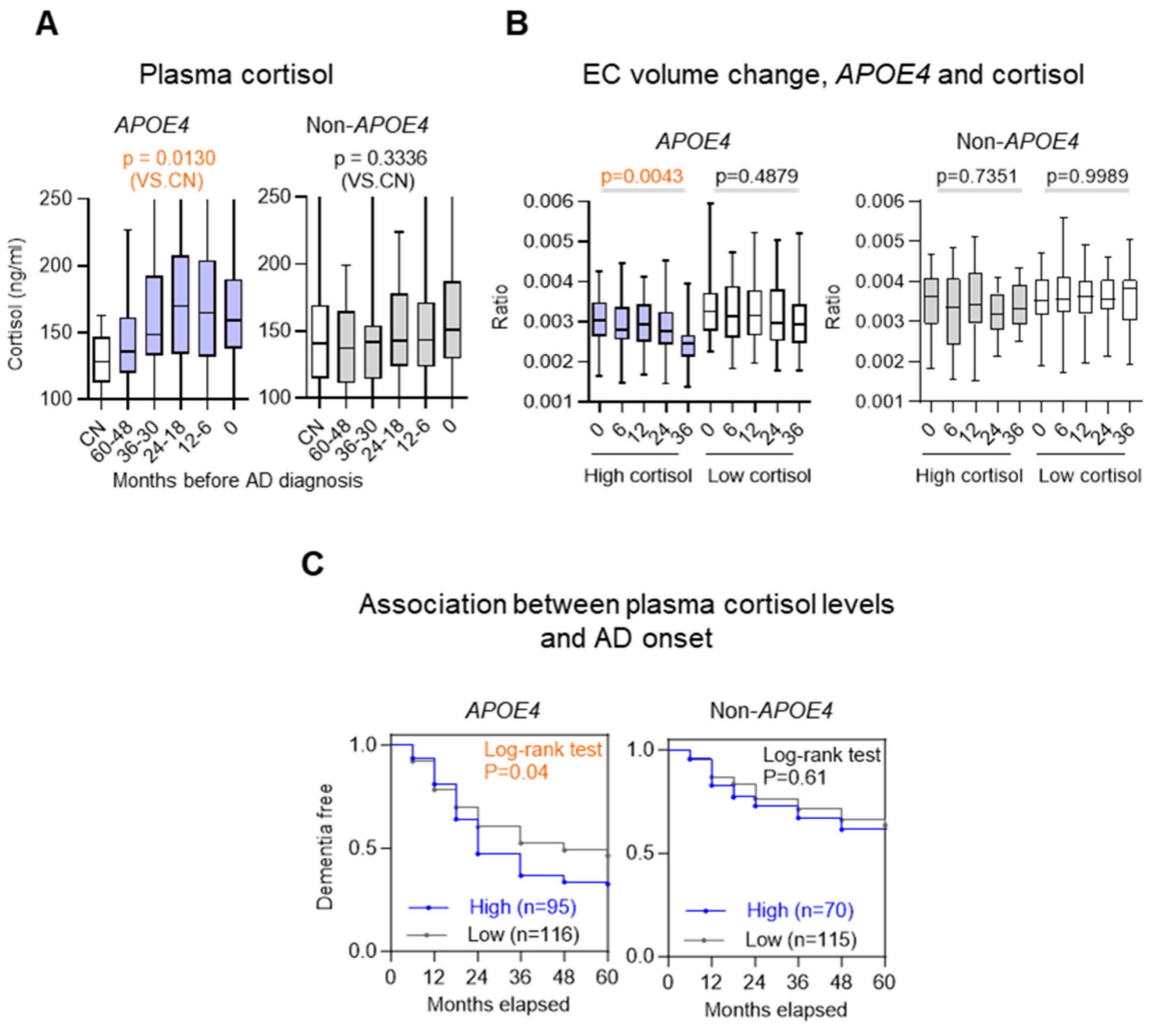
** Plasma cortisol levels as a prognostic biomarker for EC atrophy and MCI-to-AD progression in *APOE4* carriers. A.** Plasma cortisol levels were stratified by MCI-to-AD progression in *APOE4* carriers and noncarriers. Groups include cognitively normal (CN) individuals and patients categorized by time before AD diagnosis (60-48, 36-30, 24-12 and 12-6 months), with the 0-month group representing individuals already diagnosed with AD. The boxplot shows the quartile distribution of cortisol levels. **B.** EC volume changes and plasma cortisol levels during follow-up in MCI subjects were analyzed for *APOE4* carriers and noncarriers. High plasma cortisol (>150 ng/ml) was defined as levels above the MCI-stage baseline mean (visit 0), while low levels were below this threshold. EC volume was normalized by total brain volume and measured at 6, 12, 24, and 36 months, using 831 MRI data points. Statistical significance was determined by one-way ANOVA (p < 0.05). **C.** The association between plasma cortisol levels and the rate of progression from MCI to AD was evaluated in 396 subjects using a two-tailed log-rank test.

Building on the observation that *HSD11B1* SNPs are linked to both elevated plasma cortisol levels and selective EC vulnerability, we next investigated whether plasma cortisol could serve as a prognostic biomarker for EC atrophy and disease progression in individuals with *APOE4*. In *APOE4* carriers, higher plasma cortisol was observed across diagnostic stages spanning MCI to AD compared with CN (p = 0.0130 vs CN), a pattern not evident in non-carriers (Figure 3A). To further validate this relationship, we stratified MCI subjects into high- and low-cortisol groups based on baseline plasma cortisol levels and tracked EC volume changes over 36 months (Figure 3B). Among *APOE4* carriers, individuals with high cortisol showed significantly greater EC atrophy than those with low cortisol (p = 0.0043 vs. p = 0.4879). In contrast, plasma cortisol levels showed no significant association with changes in hippocampal volume, irrespective of *APOE4* status (Figure S2).

We next extending these findings to clinical outcomes. We assessed whether elevated cortisol levels predict conversion from MCI to AD. Among *APOE4* carriers, individuals with high baseline cortisol progressed to AD significantly faster than those with lower cortisol levels (χ² = 4.192, p = 0.04; HR = 1.464), whereas no significant association was found in non-carriers (χ² = 0.266, p = 0.61; HR = 1.133) (Figure 3C). Our findings are supported by prior studies demonstrating that the *APOE4* allele shows a relationship with increased EC atrophy in both AD and MCI 31, 32. Together, these results suggest that elevated cortisol has a pronounced and *APOE4*-specific impact on EC atrophy and accelerates disease progression, supporting its potential utility as a prognostic biomarker in at-risk individuals.

### APOE4 protein enhances local cortisone-cortisol conversion by upregulating *HSD11B1* in neuronal cells

To functionally validate the mechanistic model derived from our genetic, transcriptomic, biochemical, and neuroimaging analyses in two clinical cohorts, we next turned to a neuronal cell-based system to test key components of the proposed APOE4-HSD11B1-cortisol signaling pathway. Specifically, we investigated whether APOE4 protein modulates local cortisol activation in neuronal cells.

**Figure 4:**
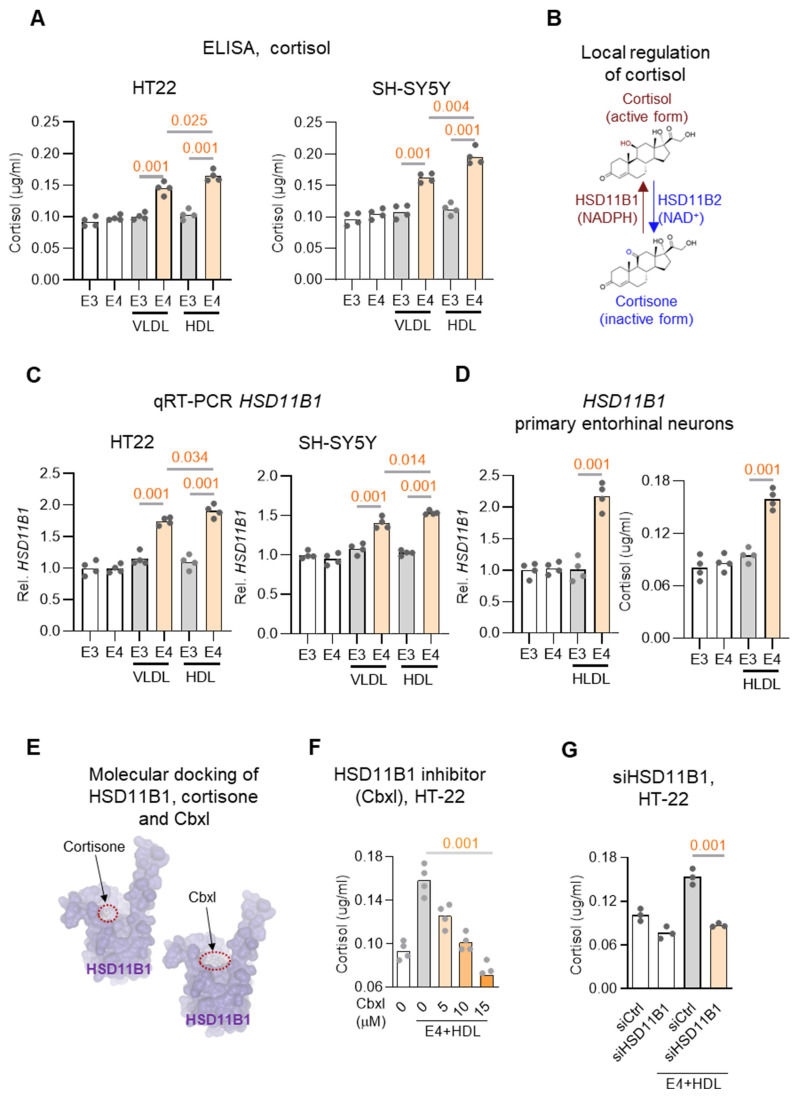
** APOE4 protein enhances *HSD11B1* expression and increases cortisol levels in neuronal cells. A.** Cortisol levels were measured in HT-22 (left) and SH-SY5Y (right) cells treated with recombinant APOE4 (E4) or APOE3 (E3) recombinant proteins. Cells were co-treated with VLDL (25 μg/mL) or HDL (25 μg/mL) plus either APOE4 or APOE3 (10 μg/mL) for 3 days, followed by cortisone (0.4 μg/mL) treatment for an additional 24 hours. Cortisol in the culture supernatants was quantified using an ELISA kit. **B.** Schematic representation of local cortisol regulation by HSD11B enzymes. **C.** qRT-PCR analysis of *HSD11B1* mRNA expression in HT-22 cells. Cells were treated under the same conditions as in (A), and *HSD11B1* mRNA levels were quantified using *GAPDH* as the internal control. **D.** APOE4/HDL induces *HSD11B1* expression and cortisol activation in primary EC neurons. Primary neurons derived from the EC were treated with recombinant APOE3 or APOE4 proteins in combination with HDL for 3 days, followed by cortisone for an additional 24 hours. Left: *HSD11B1* mRNA levels were quantified by qRT-PCR and normalized to GAPDH. Right: Cortisol levels in culture supernatants were measured by ELISA. **E.** Predicted docking models of cortisone (left) and carbenoxolone (Cbxl; right) with human HSD11B1 using SwissDock. Cortisone, the natural substrate of HSD11B1, binds within a defined pocket on the enzyme surface. Cbxl, a known HSD11B1 inhibitor, occupies a similar docking site, suggesting competitive inhibition by blocking cortisone access. Binding sites are indicated by red dashed circles. **F.** Dose-dependent reduction in cortisol levels following HSD11B1 inhibition. HT-22 cells were co-treated with APOE4 and increasing concentrations of the HSD11B1 inhibitor carbenoxolone (Cbxl; 5, 10, 15 μM) for 24 hours in the presence of cortisone (0.4 μg/mL). Cortisol levels were measured by ELISA. **G.** HSD11B1 knockdown attenuates APOE4-induced cortisol activation in HT-22 cells. HT-22 cells were transfected with siRNA (*siHSD11B1*) targeting *Hsd11b1* or a non-targeting control siRNA (*siCtrl*), followed by treatment with recombinant APOE4 in the presence of HDL for 3 days and cortisone for an additional 24 hours. Cortisol levels in culture supernatants were quantified by ELISA.

APOE is indeed a major apolipoprotein found on both Very Low-Density Lipoproteins (VLDL) and High-Density Lipoproteins (HDL) and contributes to lipid transport and neuronal metabolism 33. To investigate whether HSD11B1 acts as a downstream target of APOE4 in regulating cortisol activation in neuronal cells, two neuronal-like cell lines (HT-22 and SH-SY5Y) were treated with recombinant APOE3 or APOE4, together with VLDL or HDL, to increase the availability of steroid hormone precursors. 23, 34. Under these conditions, APOE4 significantly increased cortisol levels compared to APOE3 (Figure 4A), supporting the idea that APOE4 directly promotes local cortisol production in neuronal cells, potentially via upregulation of HSD11B1.

Since local cortisol activation is regulated by the balance between two enzymes, HSD11B1, which converts cortisone to cortisol, and HSD11B2, which inactivates cortisol to cortisone (Figure 4B) 35, we next examined whether APOE4 alters their expression. qRT-PCR analysis revealed that APOE4 significantly upregulated HSD11B1 mRNA levels, while having no significant effect on HSD11B2 expression in either cell line (Figure 4C and Figure S3A-S3B). These results indicate that APOE4 selectively enhances the expression of HSD11B1, thereby promoting local cortisol activation.

To further validate that APOE4 can modulate neuronal responses in the EC, we microdissected the EC and established primary neuronal cultures, which were subsequently treated with APOE4/HDL to assess *HSD11B1* expression (Figure 4D and Figure S4). Consistent with our observations in HT-22 and SH-SY5Y cells, APOE4/HDL markedly increased *HSD11B1* expression in EC neurons. These findings further support that APOE4 enhances *HSD11B1* expression, thereby promoting local cortisol activation.

To further validate the APOE4-induced increase in cortisol mediation is mediated by HSD11B1, we treated cells with carbenoxolone (Cbxl), an HSD11B1 inhibitor predicted by SwissDock to compete with cortisone at its binding site (Figure 4E). Cbxl treatment significantly reduced APOE4-induced cortisol levels (Figure 4F; Figure S3D). Consistently, HSD11B1 knockdown produced a similar reduction in APOE4-driven cortisol elevation (Figure 4G), further supporting a requirement for HSD11B1 in this process Together, these results provide functional evidence that HSD11B1 activity is necessary for the APOE4-driven increase in cortisol. These findings identify a key molecular mechanism by which APOE4 enhances local cortisol activation in neuronal cells, offering insight into how this pathway may contribute to neurodegeneration in AD.

### Transcription factor C/EBPβ mediates APOE4-driven *HSD11B1* expression

**Figure 5:**
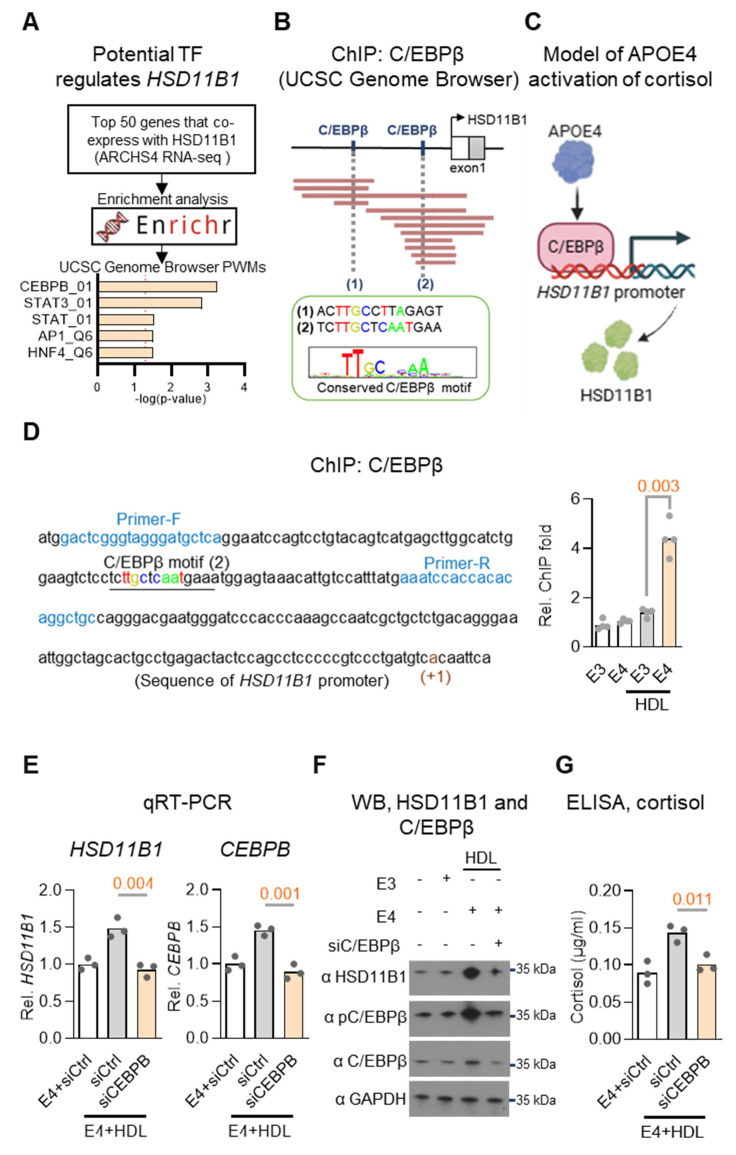
** C/EBPβ mediates APOE4-induced *HSD11B1* expression in neuronal cells. A.** Identification of potential transcriptional regulators of HSD11B1. The top 50 genes co-expressed with *HSD11B1* were identified using the ARCHS4 RNA-seq database. Enrichment analysis was performed with the Enrichr database and UCSC Genome Browser PWMs to pinpoint transcription factors potentially involved in *HSD11B1* regulation. **B.** Schematic representation of two putative C/EBPβ (*CEBPB*) binding motifs within the *HSD11B1* promoter, as identified through chromvatin immunoprecipitation (ChIP) analysis. Data were obtained from the ReMap ChIP-seq database via the UCSC Genome Browser. **C.** Proposed model illustrating how APOE4 activates C/EBPβ transcriptional activity, thereby promoting *HSD11B1* transcription. **D.** ChIP assay demonstrating C/EBPβ binding to the *HSD11B1* promoter in SH-SY5Y cells. Cells were co-treated with HDL and recombinant APOE3 or APOE4 for 3 days. ChIP-qPCR analysis quantified the enrichment of *HSD11B1* promoter fragments immunoprecipitated with anti-C/EBPβ compared to IgG controls. The promoter sequence is shown with the transcription start site (+1) marked in dark red, putative C/EBPβ binding sites underlined and highlighted in brown, and the ChIP primer sites indicated in blue. **E.** qRT-PCR analysis of *HSD11B1* mRNA levels following *CEBPB* (C/EBPβ) knockdown in SH-SY5Y cells. Cells transfected with *siCEBPB* or *siCtrl* were co-treated with HDL and APOE4 proteins, and mRNA levels for *HSD11B1* and C/EBPβ were quantified. **F.** Western blot analysis showing the effect of C/EBPβ knockdown on *HSD11B1* and C/EBPβ protein levels in SH-SY5Y cells. Cells transfected with *siC/EBPβ* or siControl were co-treated with HDL and either APOE4 or APOE3 proteins. Total cell lysates were probed with antibodies against HSD11B1, phosphorylated C/EBPβ (Thr235), total C/EBPβ, and GAPDH. **G.** ELISA quantification of cortisol levels in SH-SY5Y cells after C/EBPβ knockdown. Following transfection with* siCEBPB* or *siCtrl*, cells were co-treated with HDL and APOE4, then treated with cortisone for 24 hours. Cortisol levels in the culture supernatants were measured using a Cortisol ELISA Kit.

To investigate how APOE4 upregulates HSD11B1 expression, we sought to identify potential transcription factors involved in its regulation. We began by analyzing the ARCHS4 RNA-seq database to identify top genes co-expressed with HSD11B1^36^. To further refine this list, we conducted an enrichment analysis using the Enrichr database 37. This analysis identified C/EBPβ as a strong candidate transcription factor potentially regulating HSD11B1 expression (Figure 5A), supported by prior studies indicating that C/EBPα and C/EBPβ potential act as transcriptional regulators of *HSD11B1* 38. To further support this finding, we examined ChIP-seq datasets available through the UCSC Genome Browser and identified C/EBPβ binding sites within the *HSD11B1* promoter region (Figure 5B), reinforcing its potential regulatory role in HSD11B1 expression. Collectively, we proposed a mechanistic signaling pathway in which APOE4 activates C/EBPβ, which in turn upregulates *HSD11B1*, leading to increased local cortisol production (Figure 5C).

To experimentally validate the proposed regulatory pathway, we performed ChIP assays in SH-SY5Y cells co-treated with HDL and recombinant APOE3 or APOE4 proteins. This approach was designed to model lipid-rich conditions relevant to neuronal environments and assess isoform-specific effects of APOE on transcription factor binding. These experiments confirmed that APOE4, but not APOE3, significantly increased C/EBPβ binding at the *HSD11B1* promoter (Figure 5D). We next assessed whether C/EBPβ is required for APOE4-induced *HSD11B1* expression by knocking down C/EBPβ using siRNA in the same cell system. Combined qRT-PCR, western blot, and ELISA analyses revealed that APOE4, but not APOE3, significantly increased *HSD11B1*, C/EBPβ, phospho-C/EBPβ (T235), and cortisol levels. Importantly, silencing C/EBPβ abrogated all of these effects (Figure 5E-G), demonstrating that C/EBPβ is essential for APOE4-mediated upregulation of *HSD11B1* and subsequent cortisol activation. These findings strongly support a mechanistic pathway in which APOE4 promotes local cortisol production via activation of the C/EBPβ-HSD11B1 axis. This pathway provides a molecular explanation for our earlier observations linking APOE4 to elevated cortisol levels and EC vulnerability in AD.

Building on this mechanistic insight, we propose a model in which APOE4 promotes local cortisol activation in the EC via C/EBPβ-mediated upregulation of *HSD11B1*. This APOE4-specific signaling cascade leads to elevated cortisol levels, which may contribute to selective EC vulnerability and disease progression in AD.

## Discussion

To address the inconsistent efficacy of HSD11B1 inhibitors observed in AD clinical trials, we performed a comprehensive, multi-level analysis integrating genetic association data, transcriptomic profiling, neuroimaging, plasma cortisol measurements, and mechanistic in vitro studies. Our findings uncover a mechanistic link between *APOE4* status, HSD11B1-mediated local cortisol activation, and the selective vulnerability of the EC in AD. Specifically, we demonstrate that APOE4 enhances C/EBPβ-driven expression of HSD11B1, which catalyzes the conversion of inactive cortisone to bioactive cortisol. This APOE4 → C/EBPβ → HSD11B1 → cortisol axis may promote region-specific cortisol accumulation in the EC, thereby contributing to neurodegeneration and cognitive decline in an APOE4-dependent manner. These results provide a potential mechanistic explanation for the variability in response to HSD11B1 inhibitors and suggest that *APOE* genotype should be considered in the design and stratification of future AD therapeutic trials targeting cortisol dysregulation.

These findings suggest that HSD11B1 inhibition may not serve as a universally effective therapeutic strategy for AD, but rather may offer greater benefit in *APOE4*-positive individuals whose disease involves cortisol dysregulation in selectively vulnerable brain regions such as the EC. This genotype-dependent mechanism provides a plausible explanation for the discrepancy between the robust efficacy observed in preclinical models of HSD11B1 inhibition 14, 15 and the inconsistent outcomes reported in human clinical trials 16. Unlike preclinical models, which are typically genetically homogeneous and often lack *APOE4*, human populations exhibit substantial genetic variability, particularly with respect to *APOE* genotype, which likely influences therapeutic response. These insights highlight the importance of incorporating *APOE* genotype stratification into future clinical trial designs and support the development of *APOE4* humanized mouse models that more accurately recapitulate human AD pathophysiology.

APOE4 has been linked to activation of stress-responsive MAPK pathways, particularly p38 and JNK, which represent canonical cellular responses to proteotoxic and lipid-associated stress 34, 39-41. Together with prior evidence that p38/JNK can phosphorylate and activate C/EBPβ, the APOE4-associated increase in C/EBPβ phosphorylation suggests a potential APOE4-p38/JNK-C/EBPβ signaling route that may contribute to HSD11B1 upregulation. In addition, APOE4-driven signaling is likely modulated by its lipidation state and the local lipid-carrier environment. In the brain, APOE predominantly exists as lipidated, HDL-like particles, and variations in lipidation degree and lipid composition can influence receptor engagement and membrane-proximal signaling 42-44. Accordingly, HDL may enhance APOE4 association with neuronal membranes and amplify downstream stress signaling, providing a potential mechanistic basis for the augmented MAPK activation and HSD11B1 induction observed under lipid-rich conditions.

Moreover, the therapeutic potential of targeting HSD11B1 or upstream regulators such as C/EBPβ should be further explored in models that more closely mimic the human disease state. In parallel, future clinical trial designs could be optimized by incorporating *APOE* genotype stratification. For instance, a two-arm stratified design, in which *APOE4* carriers and non-carriers are separately randomized to receive HSD11B1 inhibitors or placebo, would enable direct comparison of treatment efficacy across genotypes. In addition to cognitive assessments, monitoring biomarkers such as plasma or CSF cortisol/cortisone ratios, as well as neuroimaging endpoints (e.g., EC volume measured by structural MRI or PET-based markers of neurodegeneration), would provide objective readouts of therapeutic response.

A key question arises from this model: if HSD11B1 inhibitors are predicted to be most effective in *APOE4* carriers, why do they also show strong efficacy in preclinical models lacking human *APOE4*? One explanation is that endogenous murine Apoe shares greater structural and functional similarity with human APOE4 than with APOE3. Evolutionarily, *APOE4* is the ancestral allele, and murine APOE retains arginine residues, pro-inflammatory properties, and glucocorticoid response profiles that resemble APOE4 45, 46. These features may render mice more responsive to HSD11B1 inhibition, thereby partially explaining the discrepancy between preclinical and clinical results. However, most AD mouse models rely on overexpressed amyloid or tau pathology, which may override subtle genotype-specific effects, and drug efficacy could reflect alternative or compensatory pathways.

Several limitations should be acknowledged. Our genetic analyses are population-based and may be influenced by confounding factors such as sex, age, or comorbidities. In addition, while cell culture experiments provide causal evidence, they do not capture the full complexity of in vivo brain environments, including neuron-glia interactions. Future studies using brain region-specific HSD11B1 knockouts and *APOE*-genotype-specific mouse models will be important to validate this pathway *in vivo*. More specifically, *APOE4*-humanized mouse models in which *HSD11B1* is either knocked out or overexpressed could provide direct evidence for the causal role of this pathway in disease progression. Such models would allow rigorous testing of whether APOE4-driven upregulation of *HSD11B1* is sufficient to accelerate EC vulnerability and tau pathology *in vivo*.

## Conclusions

This study uncovers a novel mechanistic link between the APOE4 genotype, cortisol metabolism, and the pathogenesis of AD. We show that HSD11B1-mediated glucocorticoid dysregulation contributes to entorhinal cortex vulnerability, offering a potential explanation for the persistent translational gap between preclinical findings and clinical outcomes in AD therapeutics. The identification of an APOE4-specific pathway highlights cortisol metabolism as a promising target and points toward genotype-stratified therapeutic strategies that could advance precision medicine in AD while informing broader approaches to aging and neurodegeneration.

## Supplementary Material

Supplementary figures and table.

## Figures and Tables

**Table 1:** SNPs of HSD11B1 in AD/MCI subjects with or without *APOE4* genotype.

ADNI							
		APOE4			Non-APOE4		
SNPs	A1	NMISS	STAT	P	NMISS	STAT	P
rs2235543	T	228	0.175	0.916	328	2.634	0.268
rs760951	G	228	0.072	0.965	328	3.561	0.169
rs10082169	C	228	0.155	0.926	328	2.701	0.259
rs10863782	A	228	1.212	0.545	328	3.732	0.155
rs3753519	T	222	2.192	0.334	323	0.601	0.740
rs4844908	T	228	3.756	0.153	328	1.068	0.586
rs2282738	T	227	8.351	0.015	327	0.308	0.857
rs2282739	T	228	6.182	0.045	328	0.153	0.926
rs11119328	A	228	2.130	0.345	328	1.083	0.582
rs12094117	T	228	4.414	0.110	327	0.605	0.739
rs1415542	C	228	4.110	0.128	328	0.644	0.725
rs12059226	G	228	3.690	0.158	328	0.783	0.676
rs6752	A	227	4.407	0.110	327	0.766	0.682
ROSMAP							
		APOE4			Non-APOE4		
SNPs	A1	NMISS	STAT	P	NMISS	STAT	P
rs7539852	G	323	6.136	0.031	944	4.262	0.112
rs6672256	A	325	6.052	0.038	939	4.395	0.140
rs12059226	G	324	4.957	0.067	926	5.617	0.058
rs1000283	A	322	4.884	0.064	939	4.156	0.139
rs11580294	T	325	4.860	0.053	943	0.588	0.128
rs6540546	G	327	4.720	0.127	944	2.254	0.119

#A1: A1 allele; NMISS: number of non-missing genotypes; STAT: coefficient t-statistic; P: p-value for t-statistic.

## Abbreviations

Aβ: Amyloid-beta
AD: Alzheimer's disease
ADNI: Alzheimer's Disease Neuroimaging Initiative
ANOVA: Analysis of Variance
APOE4: Apolipoprotein E ε4
BDNF: Brain-Derived Neurotrophic Factor
Cbxl: Carbenoxolone
C/EBPB / C/EBPβ: CCAAT/Enhancer-Binding Protein Beta
ChIP: Chromatin Immunoprecipitation
CI: Confidence Interval
CN: Cognitively Normal
DMEM: Dulbecco's Modified Eagle Medium
EC: Entorhinal Cortex
eQTL: Expression Quantitative Trait Locus
GTEx: Genotype-Tissue Expression
Hippo: Hippocampus
HPA: Hypothalamic-Pituitary-Adrenal
HR: Hazard Ratio
HSD11B1: 11β-Hydroxysteroid Dehydrogenase Type 1
HSD11B2: 11β-Hydroxysteroid Dehydrogenase Type 2
HT-22: Mouse Hippocampal Neuronal Cell Line
ISH: In Situ Hybridization
MCI: Mild Cognitive Impairment
MEM: Minimum Essential Medium
OR: Odds Ratio
PBS: Phosphate-Buffered Saline
PWM: Position Weight Matrix
qRT-PCR: Quantitative Real-Time Reverse Transcription-Polymerase Chain Reaction
SH-SY5Y: Human Neuroblastoma Cell Line
siRNA: Small Interfering RNA
SNP: Single Nucleotide Polymorphism
VLDL: Very-Low-Density Lipoprotein
