# Supplementary material for: Why 11β-HSD1 inhibitors show variable efficacy in Alzheimer's therapy: an APOE4-dependent HSD11B1 mechanism
Source: Theranostics. 2026 Jan 21;16(8):4113–27. doi: 10.7150/thno.126244 (PMC12905795; doi:10.7150/thno.126244)
Supplement: Supplementary file 1 — Supplementary figures and table. [file thnov16p4113s1.pdf]

Table S1. Baseline characteristics of control (CN), and MCI/AD.

ADNI				
	APOE4		nonAPOE4	
Mean ± SD	**CN**	**AD**	**CN**	**AD**
Sex (M/F)	34 / 41	92 / 61	102 / 99	85 / 42
Age	77.46 ± 0.7786	76.2 ± 0.5982	78.48 ± 0.427	78.68 ± 0.7361
CDRSB	0.8986 ± 0.2479	5.202 ± 0.291	0.4289 ± 0.08576	3.427 ± 0.2907
ADAS11	7.734 ± 0.7518	21.14 ± 0.9345	5.862 ± 0.27	14.35 ± 0.8552
MMSE	28.04 ± 0.3451	22.06 ± 0.4188	28.68 ± 0.1218	24.93 ± 0.4079
**ROSMAP**				
	**APOE4**		**nonAPOE4**	
Mean ± SD	**CN**	**AD**	**CN**	**AD**
Sex (M/F)	35/81	73/146	176/401	113/299
Age	84.22 ± 6.014	86.75 ± 4.123	84.98 ± 5.455	88.37 ± 3.067
MMSE	28.11 ± 1.717	11.233 ± 8.569	28.171 ± 1.72	14.019 ± 8.724

Notes: CN = Cognitively Normal; AD = Alzheimer's Disease; APOE4 = Presence of APOE ε4 allele; M/F = Sex (male/female); CDRSB = Clinical Dementia Rating – Sum of Boxes; ADAS11 = Alzheimer's Disease Assessment Scale – Cognitive Subscale (11 items); MMSE = Mini-Mental State Examination.

Figure S1.

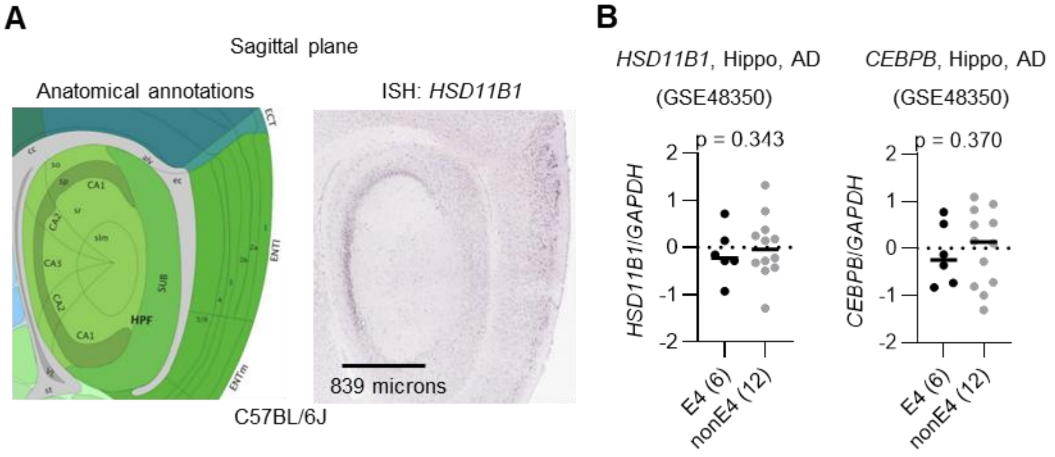

Figure S1. Differential HSD11B1 expression in the entorhinal cortex of APOE4 AD mice. (A) Sagittal plane of *HSD11B1* expression. The left panel displays anatomical annotations from the Allen Mouse Brain Atlas, the middle panel shows a Nissl-stained image from a neighboring section of the ISH image in the Allen Reference Atlas, and the right panel presents an ISH image of *HSD11B1* expression at the same slice position as panel C. Probe name: RP_071204_04_C01. **(B)** Comparison of *HSD11B1* (left panel) and *CEBPB* (right panel) expression levels in the EC between *APOE4* AD carriers and non-carriers. Microarray gene expression data from EC tissues of human AD brains were obtained from the GSE48350 dataset available at NCBI. *HSD11B1* expression was normalized using *GAPDH* as an internal control. Statistical analyses were performed using a one-tailed T-test.

Figure S2.

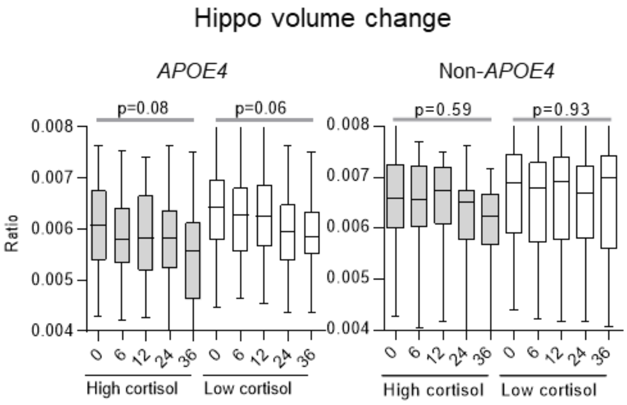

Figure S2. Hippocampus volume changes and cortisol levels during follow-up in MCI of *APOE4* and non-*APOE4* carriers. Volume measurements were normalized by dividing by whole brain volume. High cortisol levels were defined as plasma cortisol levels exceeding 150 ng/ml, while low cortisol levels were defined as levels below this threshold. Statistical significance was determined using one-way ANOVA, with a significance threshold set at $p < 0.05$.

Figure S3

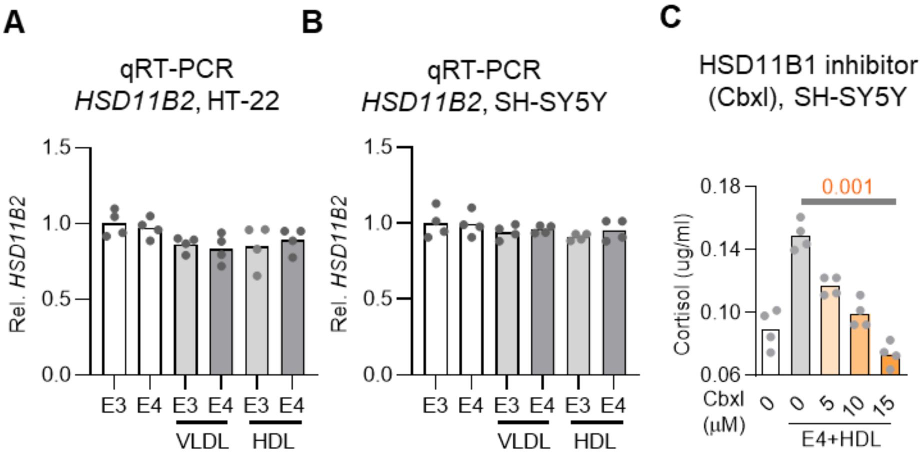

Figure S3. HSD11B1 inhibition reduces cortisol levels induced by E4+HDL treatment. (A), (B) qRT-PCR analysis of HSD11B2 mRNA levels. HT-22 (A) and SH-SY5Y (B) cells were co-treated with VLDL or HDL and either APOE4 or APOE3 proteins. HSD11B1 mRNA levels were quantified by qRT-PCR, using GAPDH as the internal control. (C) Dose-dependent reduction in cortisol levels following HSD11B1 inhibition. SH-SY5Y cells were co-treated with APOE4 and increasing concentrations of the HSD11B1 inhibitor carbenoxolone (Cbxl; 5, 10, 15 μM) for 24 hours in the presence of cortisone (0.4 μg/mL). Cortisol levels in the supernatants were measured using an ELISA kit.

Figure S4

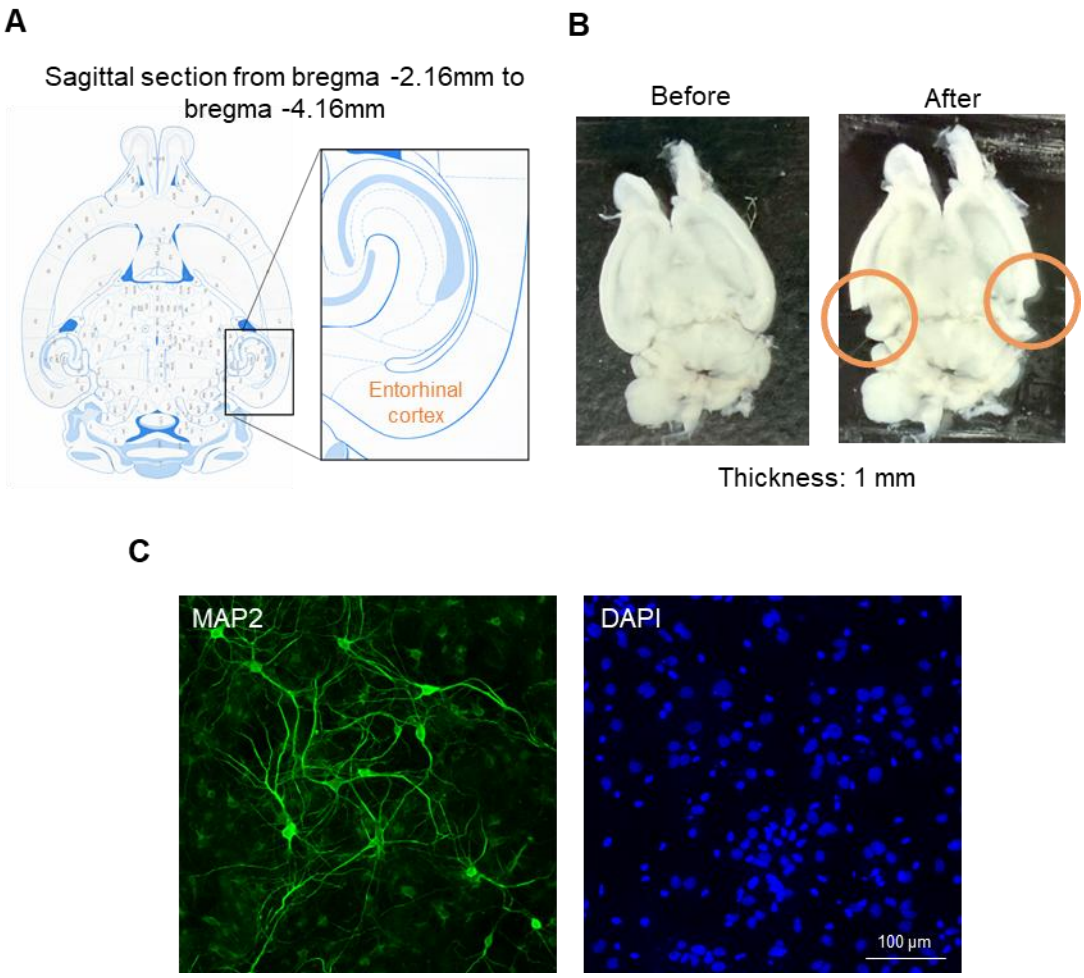

Figure S4. Microdissection of the entorhinal cortex and validation of primary neuronal cultures. (A) Schematic illustration of a mouse brain coronal section indicating the location of the entorhinal cortex (highlighted). (B) Representative images of a mouse brain before and after microdissection, showing removal of the entorhinal cortex (orange circles). (C) Representative immunofluorescence images of primary entorhinal cortical neuron cultures stained for the neuronal marker MAP-2, (green) and nuclei (DAPI, blue). Cultures were characterized by MAP-2 immunolabeling.
